# Logic + probabilistic programming + causal laws

**DOI:** 10.1098/rsos.230785

**Published:** 2023-09-27

**Authors:** Vaishak Belle

**Affiliations:** University of Edinburgh & Alan Turing Institute, Edinburgh, UK

**Keywords:** statistical relational learning, first-order logic, probabilistic programming

## Abstract

Probabilistic planning attempts to incorporate stochastic models directly into the planning process, which is the problem of synthesizing a sequence of actions that achieves some objective for a putative agent. Probabilistic programming has rapidly emerged as a key paradigm to integrate probabilistic concepts with programming languages, which allows one to specify complex probabilistic models using programming primitives like recursion and loops. Probabilistic logic programming aims to further ease the specification of structured probability distributions using first-order logical artefacts. In this article, we briefly discuss the modelling of probabilistic planning through the lens of probabilistic (logic) programming. Although many flavours for such an integration are possible, we focus on two representative examples. The first is an extension to the popular probabilistic logic programming language PROBLOG, which permits the decoration of probabilities on Horn clauses—that is, prolog programs. The second is an extension to the popular agent programming language GOLOG, which permits the logical specification of dynamical systems via actions, effects and observations. The probabilistic extensions thereof emphasize different strengths of probabilistic programming that are particularly useful for non-trivial modelling issues raised in probabilistic planning. Among other things, one can instantiate planning problems with growing and shrinking state spaces, discrete and continuous probability distributions, and non-unique prior distributions in a first-order setting.

## 1. Introduction

Automated planning is a major topic of research in artificial intelligence, and enjoys a long and distinguished history [1]. The classical paradigm assumes a distinguished initial state, comprising a set of facts, and is defined over a set of actions which change that state in one way or another. Actions are further characterized in terms of their applicability conditions, that is, things that must be true for the agent to be able to execute it, and effects, which procedurally amounts to adding new facts to a state while removing others. The scientific agenda is then to design algorithms that synthesize a sequence of actions that takes the agent from an initial state to a desired goal state.

From the early days, automated planning was motivated by robotics applications. But it was observed that the real world—or more precisely, the robot’s knowledge about the world—is almost never simply a set of facts that are true, and actions that the agent intends to execute never operate the way they are supposed to. One way to make sense of this complication is to separate the ‘high-level reasoning', in our case the planner’s search space, from the low-level sensor-motor details. On the positive side, such a move allows the plan representation to be finite, discrete and simple. On the negative side, significant expert knowledge has to go into materializing this separation of concerns, possibly at the loss of clarity on the behaviour of the system as a whole.

Incidentally, by testing the robot’s effectors repeatedly in a controlled environment, one can approximate the uncertain effects of an action in terms of a probability distribution. Similarly, based on minimalistic assumptions about the environment, expressed as a probabilistic prior, by repeated sampling, the robot can update its prior to converge on a reasonable posterior that approximates the environment [2]. To that end, probabilistic planning attempts to incorporate such models directly into the planning process. There are, to date, numerous languages and algorithmic frameworks for probabilistic planning, e.g. [3–6].

In this article, we briefly discuss the modelling of probabilistic planning through the lens of probabilistic (logic) programming. Probabilistic programming has rapidly emerged as a key paradigm to integrate probabilistic concepts with programming languages, which allows one to specify complex probabilistic models using programming primitives like recursion and loops [7]. Probabilistic logic programming aims to further ease the specification of structured probability distributions using first-order logical artefacts.

In general, probabilistic programming languages developed so as to enable modularity and reuse in probabilistic machine learning applications. Their atomic building blocks incorporate stochastic primitives, and the formal representation also allows for compositionality [7,8].

Although many flavours for such an integration are possible, we focus on two representative examples. The first is an extension to the popular probabilistic logic programming language PROBLOG, which permits the decoration of probabilities on Horn clauses—that is, prolog programs. The second is an extension to the popular agent programming language GOLOG, which permits the logical specification of dynamical systems via actions, effects and observations. The probabilistic extensions thereof emphasize different strengths of probabilistic programming that are particularly useful for non-trivial modelling issues raised in probabilistic planning. Among other things, one can instantiate planning problems with growing and shrinking state spaces, discrete and continuous probability distributions, and non-unique prior distributions in a first-order setting. More precisely, we provide an overview of the features of two systems:

— HYPE [9]: a planning framework based on *distributional clauses* [10]; and
— ALLEGRO [11]: a high-level control programming framework that extends GOLOG [12].

These two systems emphasize different strengths of probabilistic programming, which we think are particularly useful for complex modelling issues raised in probabilistic planning. HYPE can easily describe growing and shrinking state spaces owing to uncertainty about the existence of objects, and thus is closely related to BLOG models [13,14]. Since HYPE is an extension of PROBLOG [15], it stands to benefit from a wide range of applications and machine learning models explored with PROBLOG.^1^ The dynamical aspects of the domain are instantiated by reifying time as an argument in the predicates, and so it is perhaps most appropriate for finite horizon planning problems.

ALLEGRO treats actions as first-class citizens and is built on a rich model of dynamics and subjective probabilities, which allows it to handle context-sensitive effect axioms, and non-unique probability measures placed on first-order formulae. GOLOG has also been widely used for a range of applications that apply structured knowledge (e.g. ontologies) in dynamical settings [16], and ALLEGRO stands to inherit these developments. GOLOG has also been shown as a way to structure search in large plan spaces [17]. Finally, since there are constructs for iteration and loops, such programs are most appropriate for modelling non-terminating behaviour [18].

In what follows, we describe the essential formal and algorithmic contributions of these systems before concluding with open computational issues. Clearly, these two systems are not the only languages with elements of logic and probability for planning: relational Markov decision processes (MDPs) [19], first-order partially observable Markov decision processes (POMDPs) [20], and to a much lesser extent dynamic Bayesian networks over action models [21] are part of the larger family. However, these systems are built on powerful and general logical foundations: HYPE is an extension of PROBLOG, and so allows the specification of probabilistic assertions with logic programming. ALLEGRO, on the other hand, is an extension of GOLOG, which is a dialect of first-order logic with some second-order logical features. Thus, they are indicative of what is possible when probabilities and first-order logic are unified in a dynamic setting. This sets our agenda apart from proposals such as BLOG and its dynamic version too [13,14], which support some first-order features but do not permit arbitrary logical connectives and quantification.

To prepare for our discussion, let us briefly reflect on why probabilistic logic programming is a powerful paradigm.

## 2. Why probabilistic programming + logic?

Probabilistic programming is a growing field that involves representing probabilistic models as executable code [8,13,22–25]. This approach has enabled researchers to formalize, automate and scale up many aspects of modelling and inference, making them more accessible to a broader audience of developers and domain experts. By integrating modelling and inference approaches from multiple domains, this technology has also led to the development of new programmable AI systems. Probabilistic programming is widely used for formulating and solving problems in statistics and data analysis. Stochastic programming languages, such as STAN and BUGS [26], provide a formal language that gives simple, uniform and re-usable descriptions of a wide range of models, supporting generic inference techniques. Pyro, a probabilistic programming language written in Python and supported by PyTorch, enables flexible and expressive deep probabilistic modelling, unifying deep learning and Bayesian modelling [27].

Much of the mainstream discussion falls into two camps. First, for ‘conventional’ programming languages such as Python, the effort centres around support for wrapping neural computations in program code, as in the case of Pyro (and STAN to some extent). On the other extreme, support for functional programming and higher-order programming languages, and how a denotational semantics should be assigned to operational constructs in such languages, remains a challenge, especially constructs such as ‘sampling’ from a, say, normal distribution [28]. The correct sampling strategy for programmatic code, especially with loops and higher-order functions, and how these samples converge for robust inference, is also a challenging problem [24].

Amidst this, an interesting entrant is probabilistic logic programming (PLP) [10,29–35]. This concept involves the integration of probabilities into the rules of a logic program. (Although we largely focus on PROBLOG here, there is a history of interesting proposals for combining logic programming and probability, as hinted above; see [36].) Initially, the idea was to generate all possible proofs for each random choice and determine the probabilities of these ‘worlds’ [15]. However, recent advancements have led to the development of encoding strategies of programs to the task of model counting [37]. Interestingly, this problem task can be applied to many other representations, including factor graphs, relational Bayesian networks and Markov logic networks [38]. All this instantiates an incredibly powerful pipeline that offers exact inference.

In our view, four key features set probabilistic logic programming apart from other probabilistic programming techniques. Firstly, probabilistic logic programming exhibits elaboration tolerance, which means that it can easily incorporate new knowledge without having to change the entire program. Secondly, it allows for the incorporation of relational knowledge, which enables modelling of random objects and their properties. Thirdly, it can handle constraints, which makes it useful for solving optimization problems. Lastly, it supports modular non-probabilistic computation, which makes it easier to develop larger and more complex systems.

We do not mean to suggest that PLPs alleviates the problems or the representational challenges of classical probabilistic programs (PPs). To a large extent, the foci and applications of classical probabilistic programming are orthogonal to those of PLP. While it is possible to reconstruct some simple examples from PPs to PLPs, a deeper study is clearly needed. Additionally, the integration of functional artefacts and higher-order programming in the context of PLPs requires further study, despite existing work on integrating logic programming and higher-order programming [39].

Let us make these aforementioned features concrete using a few examples. Recall that declarative programming is a programming paradigm that expresses the logic of a computation without explicitly defining how the individual steps are to be executed. We describe the ‘what’ of the program rather than the ‘how to accomplish it’. What might this paradigm mean in a probabilistic context? We take this to mean that (i) the instructions and logic for decision making are fixed, but (ii) the probabilities, which are learned from data anyway, can be updated without needing to manipulate the logical instructions. Additionally, (iii) computing with these probabilities can be done without requiring input from the user. As we will show, a language like PROBLOG (but also other probabilistic logical languages) can accomplish this easily.

Suppose there is an infectious disease spreading through a population. If two people are in regular contact and one is infected, there is a 0.6 probability that the other person will also become infected. The goal is to predict the spread of the disease given a set of initially infected people and a graph of connections between individuals in the population. We might use the following program:

person(a).

person(b).

0.1::inf(X) :- person(X).

0.1::contact(X,Y) :- person(X), person(Y).

0.6::inf(X) :- contact(X, Y), inf(Y).

query(inf(_)).

This statement indicates that a person chosen at random has a 0.1 probability of being infected. Two people chosen at random have a 0.1 probability of being in contact with each other. Finally, given a randomly chosen infected person and another person in contact with that infected person, there is a 0.6 probability that the second person will become infected.

It is clear that if we need to update the probabilities or make any of them deterministic, the logical rules are unaffected. We would only need to update the numerical values. For example, to make the contagion deterministic, we may replace the fifth line above with:

inf(X) :- contact(X, Y), inf(Y).

Indeed, McCarthy’s notion of elaboration tolerance was defined as a property of a formalism that makes it convenient to modify a set of facts to accommodate new phenomena or changed circumstances. This means that the formalism can easily incorporate new knowledge without having to change the entire program. He envisoned that there are different kinds of elaboration, with the simplest being the addition of new formulae, which he called ‘additive elaborations'. Additionally, a second type of elaboration is changing the values of parameters. Although he ultimately explored these notions using a different set of examples, it is easy to see that in a very concrete sense, elaboration tolerance is seen to be supported in PLPs. Updating the probabilities or even making probabilistic assertions deterministic is easily accomplished, as seen below (example adapted from [40,41]):

0.2::inf(X) :- person(X).

0.2::contact(X,Y) :- person(X), person(Y).

inf(X) :- contact(X, Y), inf(Y).

In this case, the priors on infection and contact were increased to 0.2—probably due to new data—and the spread of infection among people in contact with each other is categorical.

If we are now to incorporate multiple sources of infection (so-called inhibition effects [40]):

0.1::inf(X) :- person(X).

0.1::contact(X,Y) :- person(X), person(Y).

0.1::groundzero(X) :- person(X).

0.6::inf(X) :- contact(X, Y), inf(Y).

0.2::inf(X) :- groundzero(X).

We need then a noisy-or structure where the parents independently influence a joint effect. All of this is accomplished without manipulating the remaining rules, and the computing of the probabilities is completely hidden from the user.

Finally, we can further contextualize influence, by allowing for, say, a distinguished group of vulnerable people, while not adjusting the weights or the rules previously constructed.

0.1::inf(X) :- person(X).

0.1::contact(X,Y) :- person(X), person(Y).

0.1::groundzero(X) :- person(X).

0.1::susceptible(X) :- person(X).

0.6::inf(X) :- contact(X, Y), inf(Y), \+ susceptible(X).

0.8::inf(X) :- contact(X, Y), inf(Y), susceptible(X).

0.2::inf(X) :- groundzero(X).

The program now additionally says that a vulnerable person has an increased chance of catching the infection.

It is worth noting that such languages could serve as an interface for other AI systems, including natural language interactions as well as deep learning models. For example, in [42], questions of the following sort can be parsed into PROBLOG programs.

### 2.1. You roll a **fair six-sided die twice**. What is the probability that the first roll shows a five and the second roll shows a six?

Here, the bold text is clearly a probabilistic model, and the rest a query. Correspondingly, the text is parsed and tokenized into a PROBLOG program of the following sort (with some additional syntax for probability and combinatorics constructs, including taking with replacement):

group(die). size(die, 6).

given(exactly(1, die, one)).

…

given(exactly(1, die, six)).

take_wr(die, rolls, 2).

probability(and(nth(1, rolls, five), nth(2, rolls, six))).

property(outcome(0), [one, two, three, four, five, six]).

Running this program then returns the answer to the question.

Conversely, in [30,43], logical reasoning is used to signal deep learning models to learn distributions that respect constraints. Underlying these models is an extremely simple but ubiquitous computational task called *weighted model counting* [44]. An extension to that task for continuous models [45] has enabled a logic-based solver strategy for a range of ‘non-logical’ (i.e. classical) probabilistic programming languages in the recent years [46–48].

## 3. HYPE

PROBLOG aims to unify logic programming and probabilistic specifications, in the sense of providing a language to specify distributions together with the means to query about the probabilities of events. As a very simple example, to express that the object *c* is on the table with a certain probability, and that all objects on the table are also in the room, we would write (free variables are assumed to be quantified from the outside):

0.6::onTable(c).

inRoom(x) :- onTable(x).

This then allows us to query the probability of atoms such inRoom(c).

A more recent extension [10] geared for continuous distributions and other infinite event-space distributions allows the head atom of a logical rule to be drawn from a distribution directly, by means of the following syntax:

h ~ D :- b1,…, bn.

For example, suppose there is an urn with an unknown number of balls [13]. Suppose we pull a ball at a time and put it back in the urn, and repeat these steps (say) six times. Suppose further we have no means of identifying if the balls drawn were distinct from each other. A probabilistic program for this situation might be as follows:

n ~ poisson(6).

pos(x) ~ uniform(1,10) :- between(1,~(n),x).

For simplicity, we assume here that the physical form of the urn is a straight line of length 10, and the position of a ball is assumed to be anywhere along this line.

HYPE is based on a dynamic extension that allows us to temporally index the truth of atoms, and so can be used to reason about actions. For example, the program

numBehind(x,t+1) ~ poisson(1) :- removeObj(x,t).

says that on removing the object *x* at *t*, we may assume that there are objects—typically one such object—behind *x*. Such programs can be used in object tracking applications to reason about occluded objects.

A common declaration in many robotics applications [2] is to define actions and sensors with an error profile, such as a Gaussian noise model. These can be instantiated in HYPE using:

pos(x, t+1) ~ gaussian(~(pos(x,t)) + 1, var) :- move(x,t).

obs(x,t+1) ~ gaussian(~(pos(x,t)), var).

The first rule says that the position of *x* on doing a move action is drawn from a normal distribution whose mean is *x*’s current position incremented by one. The second one says that observing the current position of *x* is subject to additive Gaussian noise.

As an automated planning system, HYPE instantiates a MDP [49]. Recall that MDPs are defined in terms of states, actions, stochastic transitions and reward functions, which can be realized in the above syntax using rules such as:

poss(act, t) ~ conditions(t).

reward(num, t) :- conditions(t).

As an example, imagine writing a program to instantiate an agent whose goal is to find certain types of objects (say, boxes), and move them to a specific location. (The example is adapted from [9].) Firstly, we would need to capture the frame problem [50] to say that type of the object does not change on moving it to another location:

type(X,t+1) ~ val(T) :- ~(type(X,t)) = T, not(removeObj(X)).

We might also say that boxes usually have one or more objects behind them:

numObjBehind(X,t+1) ~ poisson(1) :- ~(type(X,t)) = box,removeObj(X).

Finally, we might give a reward to the agent when it finally has the can at hand:

stop(t) :- ~ (type(X,t)) = can.

reward(20,t) :- stop(t).

reward(-1,t)

:- not(stop(t)).

To compute a policy, which is a mapping from states and time points to actions, HYPE combines importance sampling and SLD resolution to effectively bridge the high-level symbolic specification and the probabilistic components of the programming model. HYPE allows states and actions to be discrete or continuous, yielding a general planning system. Empirical evaluations are reported in [9,51].

In sum, HYPE benefits from all the advantages and modelling capabilities that have demonstrated through PROBLOG. But as can be seen, it does not quite have a distinct action language or an easy way to represent actions other than as constants indexed by time variables. This is where the situation calculus differs slightly, as it directly models a dynamic world. This allows for more flexible language for describing effects and actions, discussed below.

## 4. ALLEGRO

The GOLOG language has been successfully used in a wide range of applications involving control and planning [16], and is based on a simple ontology that all changes are a result of named actions. It is a high-level agent programming language built on the situation calculus [12], which is a dialect of first-order logic with (some) second-order logical features. An initial state describes the truth values of properties, and actions may affect these values in non-trivial context-sensitive ways. In particular, GOLOG is a programming model where executing actions are the simplest instructions in the program, upon which more involved constructions for iteration and loops are defined. For example, a program to clear a table containing an unknown number of blocks would be as follows: $([\pi x\;onTable(x)?;removeObj(x)]{)}^{*};\neg \exists x\;onTable(x)?$

Here, *π* is the non-deterministic choice of argument, semi-colon denotes sequence, ? allows for test conditions, and * is unbounded iteration. The program terminates successfully because the sub-program before the final test condition removes every object from the table.

As argued in [16], the rich syntax of GOLOG allows us, on the one hand, to represent policies and plans in an obvious fashion; for example: $\left(a_{1};\ldots ;a_{n};P?\right)$ ensures that the goal *P* is true on executing the sequence of actions. However, the syntax also allows open-ended search; for example: (while ¬P πa. a) tries actions until *P* is made true. The benefit of GOLOG then is that it allows us to explore plan formulations between these two extremes, including partially specified programs that are completed by a meta-language planner.

ALLEGRO augments the underlying ontology to reason about probability distributions over state properties, and allow actions with uncertain (stochastic) effects. In logical terms, the semantical foundations rest on a rich logic of belief and actions. Consequently, it can handle partial probabilistic specifications. For example, one can say *c* is on the table with a certain probability as before: pr(onTable(c))=0.6, but it is also possible to express the probability that there is an object on the table without knowing which one: pr(∃x onTable(x))=0.6. We can go further and simply say that there is a non-zero probability of that statement: pr(∃x onTable(x))>0, which means that any distribution satisfying the formula is admissible. Such a feature can be very useful: for example, in [52], it is argued that when planning in highly stochastic environments, it is useful to allow a margin of error in the probability distributions defined over state properties.

To model the case of Gaussian error models, actions with uncertain effects are given a general treatment. For one thing, the effects of actions are axiomatized using the notion of successor state axioms which incorporate Reiter’s solution to the frame problem [12]. So, for example, changing the position of an object using a move action can be expressed as:

pos(x,do(a,s))=u≡(a=move(x,y)∧pos(x,s)=u+y)∨(a≠move(x,y)∧pos(x,s)=u).

This says that if the action of moving *x* was executed, its position (along a straight line) is decremented by *y* units, and for all other actions, the position is unaffected. To deal with uncertain effects, we will distinguish between what the agent intends and what actually happens. That is, let *move*(*x*, *y*, *z*) be a new action type, where *y* is what the agent intends, and *z* is what happens. Then, the successor state axiom is rewritten as follows:

pos(x,do(a,s))=u≡(a=move(x,y,z)∧pos(x,s)=u+z)∨(a≠move(x,y,z)∧pos(x,s)=u).

The story remains essentially the same, except that *z* determines the actual position in the successor state, but it is not in control of the agent. A Gaussian error profile can be accorded to this action by means: l(move(x,y,z),s)=gaussian(z;y,var). That is, the actual value is drawn from a Gaussian whose mean is the intended argument *y*. Analogously, attributing additive Gaussian noise in a sensor for observing the position is defined using: l(obs(x,z),s)=gaussian(z;pos(x,s),var). That is, the observation *z* is drawn from a Gaussian whose mean is the actual position of the object *x*.

As hinted above, as an extension to GOLOG, the syntax of ALLEGRO is designed to compactly represent full or partial plans and policies in a general way. Previously, GOLOG programs have been shown to control the plan search space [17], and recent work has shown that probabilistic action programs are a more compact way to represent recursive policies. On termination, ALLEGRO programs can be tested for, say, the (subjective) probability that the goal is believed to hold.

For example, imagine you wish to get a robot with noisy sensors and effectors close to a wall. The robot would move some distance, but because the action is noisy, it would need to repeatedly sense to ensure it did actually get closer. This sense-act loop would repeat until it believes with high probability that it is actually close to the wall. An ALLEGRO program for such a protocol might look like this:

WHILE Pr(distance <= 2)

  forward(1) | sonar;

ENDWHILE

So we recur on either moving or sensing (declared using the non-deterministic branch operator) until the robot believes the distance to the wall is less than, say, 2 units. Here, the action forward(1) denotes an action *forward(x,y)*, where the intended argument is (*x* = 1), but the actual argument is, as discussed above, unknown to the agent. Since the well-defined terms in the language will need a distinct value for *y*, we will need the value for *y*, as determined by, say, nature or the environment. However, the belief of the agent will not be affected by this value, since the value is not observed. So any randomly chosen value for *y* would also suffice to understand program correctness.

Somewhat analogously, sonar might denote the action *sonar(z)*, where *z* is the value read on the sensor. But unlike the actual argument for actions above, the value read does matter to the beliefs of the agent, because it adjusts its beliefs based on what was observed. So we need to incorporate these read values to ascertain if the program terminates.^2^ The semantical foundations of ALLEGRO incorporating these ideas was established in [11] with a discussion on its empirical behaviour.

The above program fully specifies the behaviour, but at the same time, it is capturing a very simple example. A more complex problem setting might involve unspecified sub-programs (which might involve planning), as discussed in [16]. Alternatively partially specified programs might be used to define and constrain the search space, as seen in [17].

## 5. A qualitative comparison

Before concluding the article, it is an interesting exercise to briefly compare the features and properties of the two formalisms. Of course, readers may have the impression that such a comparison would be richer with an empirical evaluation, perhaps by benchmarking the two formalisms against each other in a set of domains, to compare their computational performance. Although this is feasible for a particular problem space (and worth considering for the future), the languages are not fully comparable, as the discussion below will demonstrate.

### 5.1. Static modelling

We refer to static modelling here to mean capturing a probability distribution over a finite set of random variables. Given a number of atoms in a propositional language (for simplicity), assume atoms are accorded probabilities and these are taken together with non-probabilistic definitive clauses, of the form *h* ← *b*_1_, …, *b*_*k*_. For example, here is a PROBLOG program for the burglary-earthquake-alarm Bayesian network example by Pearl [56]:

0.1::b.

0.2::e.

a :- b.

a :- e.

query(a).

That is:

— *b* is a proposition standing for burglary and has a probability of 0.1 of occurrence;
— *e* is a proposition standing for earthquake and has a probability of 0.2 of occurrence;
— *a* ← *b* is a non-probabilistic rule that says that burglary can trigger the alarm, captured using proposition *a*; and
— *a* ← *e* is a non-probabilistic rule that says that earthquake can trigger the alarm too.

The reader may now construct a set of possible worlds to work out the marginal probabilities. With three propositions, we have eight possible worlds in principle. However, there are some caveats to how we read these atoms and rules in PROBLOG. The atoms for which we have provided probabilities are called *probabilistic facts* and should be treated separately from the heads of rules. In fact, the rules would be used in conjunction with each of the possible worlds (defined purely over the probabilistic atoms) to determine if the head can be derived. Based on that, the success probability of the head is calculated.

Putting that together, we have four worlds over the probabilistic facts *b* and *e*. The probability of the worlds is obtained by taking the product of the probabilities of literals built from *b* and *e*, with the understanding that the probability of any proposition *p* and its negation must sum up to 1. So, for example, in a world where *b* is true but *e* is false, we would obtain the probability of 0.1 × (1 − 0.2) which is 0.08. By extension we have

1. the world b∧¬e with probability 0.08 as just determined;
2. the world b∧e with probability 0.1 × 0.2 = 0.02;
3. the world ¬b∧e with probability (1 − 0.1) × 0.2 = 0.18; and
4. finally, the world ¬b∧¬e with probability (1 − 0.1) × (1 − −0.2) = 0.72.

To now calculate the probability of triggering the alarm, we look at each possible word together with the non-probabilistic rules and see if *a* is entailed. It is easy to see that this only happens with the first three worlds: here, either burglary or alarm is true and so it immediately follows that *a* holds. Thus, the probability of triggering the alarm is 0.28. See [37] for the full semantic treatment.

Thus, the semantics of logic programs seem particularly conducive to modelling Bayesian networks [44]. By contrast, the semantics of ALLEGRO is based on probabilities on first-order structures. So it is not quite the exact same set of formulae needed to replicate that network. We need to slightly adapt it. For one thing, there is nothing in semantics that suggests that certain atoms should not have probabilities, as it is implicitly assumed in the semantics of PROBLOG programs. So, for instance, we could use the following formulae in ALLEGRO for the network:

— Pr(b)=0.1;
— Pr(e)=0.2;
— a≡b∨e and Pr(a)=1.

In ALLEGRO, then, because there are three propositions we indeed have eight possible worlds in principle. The last formula, however, ensures that worlds where *b* and *e* are false but *a* is true are impossible.

If we now contrast this with the four possible worlds we had for PROBLOG, we can indeed see that it is the same set of truth-settings for *b* and *e* (the first three) that would be considered for the probability of *a*. The fourth world ¬b∧¬e cannot be one where *a* is true, as established. However, the calculation of the probabilities of worlds is obtained in a different manner, but amounting to the same specification. The formulae, for example, state that all the worlds where *b* is true need to be assigned a total probability of 0.1. This essentially means the first and second worlds, (1) and (2) above, need to have their probabilities sum to 0.1. Likewise, the set of all worlds where *e* is true needs to be assigned a total probability of 0.2. So the second and third worlds, (2) and (3) above, need to have their probabilities sum to 0.2. By working through these, it can be shown that the probability of the alarm being triggered is still 0.28.

The fact that the formulae need to be mapped this way between the formalisms may seem strange at first glance, but it is not surprising. A translation of PROBLOG programs to probabilistic propositional theories [37] relies on these kinds of steps to assign probabilities to propositional worlds, which as we just established, needs to take all the propositions in the language into account.

Of course, it is entirely reasonable to expect modelling languages to be as suitable as needed for the problem at hand. Clearly, when capturing Bayesian networks and other related types of graphical models, PROLOG and HYPE are much more straightforward than, say, specifying probabilities over first-order formulae. For the latter, we need to implicitly determine how these probabilities correspond to a Bayesian network or some other factored representation [57].

However, given that PROBLOG programs are then translated into probabilistic propositional theories, one could imagine that there is a more natural modelling language for users who prefer graphical models. Then an intermediate step would translate it into an ALLEGRO model so that the initial knowledge can be used with a rich theory of actions. Moreover, there is one case where the static knowledge base between these formalisms may not require much reworking. Consider probabilistic databases [58]: the simplest setting is that of *tuple-independent* probabilistic databases, where each proposition is assigned a distinct probability, and assumed to be probabilistically independent from others. In such a setting, the representation in both formalisms would be virtually identical because the probability of each world is essentially specified explicitly: it is obtained from the product of the probability of the propositions true at the world.

There is one aspect in which the formalisms differ deeply. Logic programs allow the use of inductive definitions [59]. For example, we might use the following two formulae to capture an inductive definition for paths in a graph:

— ∀x,y(E(x,y)⊃P(x,y)); and
— ∀x,y,z(E(x,y)∧P(y,z)⊃P(x,z)).

This says that every edge is a path, and if there is an edge between nodes *x* and *y* and a path from *y* to *z*, then there is also a path from *x* to *z*.

ALLEGRO is based on the situation calculus, which allows for some second-order features, including an inductive definition for the set of situations [12]. However, it is rarely the case that inductive definitions are provided as part of the initial knowledge base. (Nonetheless, there is some work regarding this in the literature [60].) Here too, PROLOG and HYPE may seem like more attractive formalisms if one is mostly concerned with reasoning about static knowledge.

### 5.2. Observations and actions

We already discussed how these languages do allow the modelling of dynamics, but mainly as captured by dynamic Bayesian networks, hidden Markov models, MDPs, POMDPs and other related frameworks [29,36]. This is achieved by introducing a time argument for the predicates, and reasoning about a certain number of time steps.

Nonetheless, such an approach makes these languages more limited compared with dynamic languages like the situation calculus. In the situation calculus, not only is it possible to have durative actions where time is an additional argument [61], but the dynamics of states can be captured directly using named actions. As a result, it becomes natural to capture numerous planning languages [12].

The situation calculus has also been extended to reason about decision-theoretic models [62] and relational extensions to POMDPs [20]. Its first-order nature also makes it a candidate for reasoning about unbounded numbers of objects and unbounded time sequences. For example, it is possible to ask *projection queries* of the following sort:

— Is *ϕ* true after doing actions *a*_1_ to *a*_*k*_?
— Was *ϕ* true at every step of the sequence, or conversely was *ϕ* made false at any point between the start and the end of the sequence?
— Will *ϕ* be true for every sequence executed from here on?

Thus, not only can one write programs in the language, but one can also verify the properties of these programs directly within the language [63].

It is also worth noting that the action types themselves are probably more powerful than what can be provided in a formalism such as a dynamic Bayesian network [64]. For example, in the context of the Bayesian network used previously on the alarm getting triggered, one can write actions that can potentially change the edges between nodes in an arbitrary fashion and add new nodes. We can also reason about the ramifications and indirect effects of actions [65].

### 5.3. Synthesis versus policy execution

As was made clear from the presentation of HYPE, that language is primarily for synthesizing a policy. That is, it specifies actions and effects and the noise models, and the purpose of the formalism is to compute a policy from that specification. We can contrast that to ALLEGRO in two ways. Firstly, belief-based programs are policies in themselves. So, executing the program is tantamount to executing a policy for a POMDP under the appropriate representational considerations [66]. But of course, such a policy could also be synthesized. There is considerable work on the induction of recursive program-like structures [67]. Thus, the situation calculus provides a comprehensive framework to reason about different sorts of policies and verify their properties.

### 5.4. Incomplete and partial specifications

Perhaps all of these different dimensions are essentially a statement about the differences in expressiveness. While PROBLOG and HYPE mainly provide a framework for specifying probabilistic models, the situation calculus provides a language for reasoning about probabilistic knowledge and its dynamics more generally. For instance, it is possible to express multiple belief distributions, specify programs either completely or partially, and combine probabilistic knowledge with non-probabilistic, incomplete knowledge (which then necessitates the need for multiple distributions) [68].

All of this means that the situation calculus language is far more expressive because it cannot be reduced to a propositional model. Recall that PROBLOG reduces its inference to a weighted propositional formula, whereas the situation calculus corresponds to a modal first-order logic [69].

Consequently, due to this expressiveness, it can be shown that verifying properties in the full language is highly undecidable [63]. In the case of ALLEGRO, verifying properties would correspond to checking the properties of possibly infinitely many POMDPs.

Of course, the full expressiveness need not be embraced. Depending on the application, we might choose a fragment to work with, perhaps a fragment that closely resembles PROBLOG and HYPE. This is the argument the knowledge representation community have been making all along [12,16]: sometimes it is useful to have a more expressive language to study the properties of the desired problems and then we would choose a restricted fragment to build the domain application at hand.

## 6. Conclusion

Automated planning is often deployed in an application context, and in highly stochastic and uncertain domains, the planning model may be derived from a complex learning and reasoning pipeline, or otherwise defined over non-trivial state spaces with unknowns. In this article, we reported on two probabilistic programming systems to realize such pipelines. Indeed, combining automated planning and probabilistic programming is receiving considerable attention recently, e.g. [14]. These languages are general purpose, and their first-order expressiveness can not only enable a compact codification of the domain but also achieve computational leverage.

One of the key concerns with the use of probabilistic programming and stochastic specifications generally is that most systems perform inference by Monte Carlo sampling. As is well known, one is able to only obtain asymptotic guarantees with such methods, and moreover, handling low-probability observations can be challenging. In that regard, there have been recent logical approaches for inferring in mixed discrete-continuous probability spaces with tight bounds on the computed answers [45,46,70]. Since HYPE, ALLEGRO and many such systems use probabilistic inference as a fundamental computational backbone, the question then is whether the aforementioned approaches can enable robust planning and programming frameworks in stochastic domains.

There may also be added value in finding deeper connections with conventional probabilistic programming languages. For example, quite a bit of work has been done on trying to learn in such conventional languages [71]. Additionally, deep learning techniques can be integrated as computational primitives in such languages, as seen in Pyro. It would be interesting to explore whether such ideas can be incorporated into HYPE and ALLEGRO to unify first-order probabilistic reasoning and learning.

## Data Availability

This article has no additional data.

## Footnotes

1
dtai.cs.kuleuven.be/problog

2 It might be possible, of course, to define a notion of program correctness that works for any observed value, in a style similar to the offline computation of policies [53–55]. We gloss over this issue for now.
